# Comparing two approaches for estimating the causal effect of behaviour-change communication messages promoting insecticide-treated bed nets: an analysis of the 2010 Zambia malaria indicator survey

**DOI:** 10.1186/1475-2875-13-342

**Published:** 2014-08-30

**Authors:** Marc Boulay, Matthew Lynch, Hannah Koenker

**Affiliations:** Johns Hopkins University Center for Communication Programs, Baltimore, Maryland USA

**Keywords:** Malaria, Behaviour-change communication, Insecticide-treated bed net, Long-lasting, Insecticidal-treated bed net, Zambia

## Abstract

Over the past decade, efforts to increase the use of insecticide-treated bed nets (ITNs) have relied primarily on the routine distribution of bed nets to pregnant women attending antenatal services or on the mass distribution of bed nets to households. While these distributions have increased the proportion of households owning ITNs and the proportion of people sleeping under an ITN the night prior to the survey, the role that behaviour-change communication (BCC) plays in the use of ITNs remains unquantified.

This paper uses two analytic approaches, propensity score matching and treatment effect modelling, to examine the relationship between exposure to the BCC messages and the use of a bed net the previous night, using the 2010 Zambia Malaria Indicator Survey (MIS).

When matched on similar propensity scores, a statistically significant 29.5 percentage point difference in ITN use is observed between exposed and unexposed respondents. Fifty-nine per cent of unexposed respondents reported sleeping under an ITN the previous night, compared to 88% of the exposed respondents. A smaller but similarly significant difference between exposed and unexposed groups, 12.7 percentage points, is observed in the treatment effect model, which also controls for the number of bed nets owned by the household and exposure to malaria information from health workers.

Using either approach, a statistically significant effect of exposure to BCC messages on a woman’s use of an ITN was found. Propensity score matching has the advantage of using statistically-matched pairs and relying on the assumption that given the measured covariates, outcome is independent of treatment assignment (conditional independence assumption), thereby allowing us to mimic a randomized control trial. Results from propensity score matching indicate that BCC messages account for a 29-percentage point increase in the use of ITNs among Zambian households that already own at least one ITN.

These analyses serve to illustrate that BCC programmes can contribute to national programmes seeking to increase the use of ITNs inside the home. They also offer a viable approach for evaluating the effectiveness of other BCC programmes promoting behaviour that will reduce malaria transmission or mitigate the consequences of infection.

## Background

Insecticide-treated bed nets (ITNs) are widely regarded as an effective tool for preventing the transmission of malaria [1, 2]. Over the past decade, efforts to increase the use of ITNs have relied primarily on the routine distribution of bed nets to pregnant women attending antenatal services or on the mass distribution of bed nets to households. While these distributions have increased the proportion of households owning ITNs and the proportion of people sleeping under an ITN the night prior to the survey, there are several reasons for questioning the sole reliance on distribution approaches for increasing ITN use. First, a substantial number of households with an ITN continue to report that they do not use them [3–6]. Second, qualitative studies in many settings have consistently uncovered attitudes and beliefs unrelated to access and availability that contribute to decisions to not sleep under a bed net [7–10]. Third, there are anecdotal reports from the field that households sometimes repurpose usable ITNs for other household uses.

These findings suggest that behaviour-change communication (BCC) programmes may have a role to play in changing these attitudes and promoting the effective use of ITNs. An extensive body of literature provides evidence of the effectiveness of BCC for increasing a range of positive health behaviours [11, 12]. Despite this evidence, the widespread use of BCC approaches for promoting ITNs is hampered by continued scepticism regarding their effectiveness.

Much of this scepticism stems from the challenge of evaluating BCC approaches using trials that randomly allocate individuals to treatment or control conditions [13]. Rather than random allocation, individual preferences and opportunities often determine whether a person listens to a programme message on a mass media channel or participates in a group activity within the community. Since the characteristics that influence an individual’s exposure to a programme message may also influence the behaviour targeted by that message, any observed difference in the behaviour between the treatment and control groups is potentially due to the confounding effects of these additional factors.

However, there is a growing recognition within the public health community of alternative evaluation designs that allow for causal attribution using observational data in situations where the random allocation is not feasible [14–16]. One approach, propensity score matching, uses exogenous background variables to create statistically identical intervention and control groups conditioned on those background variables. In this approach, exogenous background variables are regressed on a binary treatment exposure variable to calculate individuals’ propensity to be exposed to the intervention. Using the propensity scores to match similar exposed and unexposed individuals, this approach is able to estimate the causal effect of the treatment on the outcome of interest, assuming two conditions are true. First, this approach assumes that the variables used to construct the propensity score account for all of the potential confounding between exposure to the intervention and the outcome behaviour of interest (conditional independence assumption). And, if the first assumption is true, the matching of exposed and unexposed individuals with similar propensity scores removes all differences related to these background variables between the two groups.

While the use of propensity scores is a conceptually appealing analogue to experimental designs, there are two challenges related to its use in BCC evaluations. First, it is often difficult to assume that all potential confounders have been accounted for in the propensity score equation. Second, the need to use many variables to construct an adequate propensity score often makes it difficult to balance each of these variables between the exposed and unexposed groups after matching. This second challenge was illustrated by Bowen [17], whose use of the propensity score approach to evaluate malaria-related BCC activities in Cameroon was clouded by the differences between exposed and unexposed groups that remained after matching.

Treatment effect models [18, 19] offer an alternative approach for assessing causal effects of BCC programmes. Similar to the propensity score approach, these models use exogenous background characteristics to predict individuals’ likelihood of being exposed to the intervention. However, treatment effect models differ from the propensity score approach by using the predicted exposure to the intervention as an instrumental variable in a simultaneous equation that predicts the outcome behaviour of interest. Treatment effect models offer two advantages over the propensity score matching approaches. First, by estimating the treatment and outcome equations simultaneously, treatment effect models are able to assess, and account for, the potential for unmeasured factors to confound the relationship between treatment and outcome by calculating the extent to which the error terms in the two models are correlated. Second, treatment effect models support the inclusion of additional influences on the outcome of interest beyond the treatment. However, these selection models are based on assumptions of the joint distributions of the error terms from the two models that are not always easy to justify [20].

Using data from the 2010 Zambia Malaria Indicator Survey (MIS), this paper compares the two approaches for evaluating the effectiveness of a BCC intervention on the use of ITNs. Given the differing strengths and limitations of the two approaches, similar findings between them will provide stronger evidence of the effectiveness of the BCC programmes. This dataset was selected for this analysis due to the quality of the data and the strength of the BCC activities prior to the data collection period. Prior to the 2010 MIS fieldwork, Zambia had an ongoing national mass and print media campaign for net use, as well as community-level campaign using interpersonal and community-based approaches [21].

## Methods

### Data

Data for this analysis were collected as part of the 2010 Zambia MIS. The survey used a cluster-based sample design to select a representative sample of 4,500 households from 180 clusters throughout the country. A total of 4,361 selected households agreed to participate in the survey, for a 97% acceptance rate. In each household, all women between the ages of 15 and 49 years of age were eligible to be interviewed and a total of 4,211 women consented to participate in the interviews. Fieldwork for the survey occurred in April and May 2010, which corresponds with the beginning of the dry season in Zambia.

Since these analyses were intended to explore the role of BCC messages on decisions to use an ITN the night prior to the interview, households without any ITN were excluded from the analysis. Seventy per cent of all households (n = 3,071) reported owning at least one ITN. Among households owning at least one ITN, 3,380 women were interviewed and were included in the current analysis.

### Variables

The outcome variable for this analysis was a binary variable recording whether each woman in the sample reported sleeping under any ITN the night prior to the survey. This outcome, rather than children’s or all household members’ use of ITN, was selected to most closely link exposure to BCC messages with the behaviour. Each woman’s reported ITN use the previous night was obtained from the household net roster, a standard component of the MIS.

To measure exposure to BCC messages, all women were asked whether they had ever heard or seen any messages about malaria and, if so, how many months ago did they hear or see these messages. These questions were asked only in the women’s questionnaire, preventing further analysis by gender of the respondent. For this analysis, the criteria for exposure was limited to women who reported hearing or seeing any malaria messages in the past six months and also cited at least one specific channel: television or radio, in the newspaper, on posters or billboards, or from peer educators and drama groups.

Seven exogenous variables were included as potential predictors of BCC message exposure to calculate both the propensity score for matching and in the treatment effect model. These included the individual-level characteristics of age of the respondent, measured in individual years; education of the respondent, coded as a three-level variable measuring no education, primary only, or more than primary; and, a binary variable coding whether the woman has a child under the age of six years old. Household-level variables also considered to potentially influence BCC message exposure included: wealth quintile, measured using a standard household possession index; a binary variable measuring residence in an urban or rural area; a binary variable measuring whether or not the household is in a district scheduled to receive indoor residual spraying (IRS); and, province of residence. To maximize the sample size, provinces were grouped into four geographic zones: a) Central and Copperbelt; b) Eastern, Northern and Luapula; c) Lusaka; and, d) Western, Southern and North-Western.

Finally, two additional predictors of ITN use were considered. First, the number of ITNs owned by the household was expected to be positively associated with a woman’s likelihood of sleeping under an ITN the previous night. Second, discussion of malaria with a health worker was expected to have an effect on ITN use independent of exposure to BCC messages about malaria.

### Analysis

The paper uses two analytic approaches, propensity score matching and treatment effect modelling, to examine the relationship between exposure to the BCC messages and the use of an ITN the previous night. Morgan and Winship [22] and Hutchinson and Wheeler [20] provide a thorough overview of both approaches.

For both the propensity score matching and the treatment effect model, the same probit regression equation was used to estimate the likelihood of exposure to BCC messages conditioned on the seven exogenous variables described in the section above. The predicted probabilities of message exposure derived from this equation served as each respondent’s propensity score. In the PSM analysis, nearest neighbour-matching with replacement was used to match exposed and unexposed respondents with similar propensity scores and t-tests for the equality of means to assess the similarity between the matched treatment and control groups for each of the exogenous background variables. A bootstrap approach was used to calculate the standard error and significance of the difference in ITN use between the intervention and treatment groups. This analysis used the psmatch2 command available within Stata 12 [23].

The treatment effect model used the biprobit regression command within Stata 12. This command simultaneously estimates two probit regression models and is appropriate for situations where both the treatment and outcome are measured as binary variables. The predicted probabilities of message exposure derived from the first equation were used as an instrumental variable predicting ITN use within the second equation. The estimated effect of the instrumental variable on ITN use provided the comparison of the average treatment effect between the intervention and control groups. The adjusted probabilities of ITN use between exposed and unexposed individuals were calculated based on the estimated effect of the instrumental variable to facilitate comparisons of the effect of exposure to the crude, unmatched, and matched outcomes.

## Results

### Overview of the main variables

Table 1 summarizes the variables included in the current analysis. The outcome behaviour, use of an ITN the night prior to the survey, was relatively common in the sample. Overall, 77.6% of respondents living in a house that owned at least one ITN reported sleeping under an ITN the preceding night.

**Table 1:** **Frequency distributions for variables included in the analysis**

Variable	Freq (%) (n = 3,263)
Slept under an ITN the previous night	
No	731 (22.4)
Yes	2,532 (77.6)
Exposed to BCC messages through mass media and/or community-based channels	
No	2,498 (76.6)
Yes	765 (23.4)
Age (in years)	
15-24	934 (28.6)
25-34	1,230 (37.7)
35-49	1,099 (33.7)
Education	
None	817 (25.0)
Primary	1,568 (48.0)
Secondary or higher	878 (26.9)
Province of residence	
Central, Copperbelt	433 (13.3)
Eastern, Northern, Luapula	1,342 (41.1)
Lusaka	354 (10.9)
Western, Southern, North-Western	1,134 (34.8)
Has a child under the age of 6 years	
No	1,166 (35.7)
Yes	2,097 (64.3)
Wealth quintile	
Poorer three quintiles	1,597 (48.9)
Wealthier two quintiles	1,666 (51.1)
Live in a district that received IRS	
No	376 (11.5)
Yes	2,887 (88.5)
Live in an urban area	
No	2,630 (80.6)
Yes	633 (19.4)
Number of nets in household	
1	413 (12.7)
2	1,168 (35.8)
3+	1,682 (51.55)
Talked to a health worker about malaria	
No	1,208 (37.2)
Yes	2,055 (63.0)

The primary independent variable, reported exposure to BCC messages about malaria in the previous six months and mention of at least specific channel, was relatively uncommon. Only 23% of the sample reported hearing or seeing any messages about malaria in the six months prior to the survey and were also able to spontaneously name the channel. Approximately two-thirds of those exposed to any messages about malaria reported hearing or seeing them on mass media channels, while one-third reported hearing or seeing malaria messages at community events.

The two other potential predictors of ITN use included exposure to malaria information from health workers and number of bed nets of any kind owned by the household. Slightly more than 60% of respondents reported that they had talked about malaria with a health worker in the previous six months. Over half of all respondents in the sample reported that their household owned three or more bed nets of any kind, while another 36% reported that their households owned two bed nets. Only 13% of respondents reported that their household only owned one bed net. Nearly 90% of nets in the overall sample were ITN or LLIN; only 630 out of 5732 were untreated.

The largest proportion of respondents included in the analysis were between the ages of 25 and 34 years (38%), had a primary education (48%), had a child under the age of six years (64%), were living in a district that had received IRS for malaria prevention (89%), and were living in a rural area (81%). Since households that did not own any ITN were excluded from the analysis, the proportion of respondents from the upper two wealth quintiles was slightly higher than the proportion of respondents from the bottom three wealth quintiles. Most respondents were from the combined Eastern, Northern and Luapula set of provinces (41%).

### Propensity score matching

Six of the seven variables included in the probit regression model to predict exposure and calculate each respondent’s propensity score were significantly related to exposure. Older respondents between the ages of 35 and 49 years and those with more education were more likely to have heard or seen BCC messages about malaria on the mass media or at community events. Respondents in the upper two wealth quintiles, with a child under the age of six years, and living in a district that received IRS were significantly less likely to have heard or seen BCC messages about malaria. Controlling for other background characteristics, respondents not living in the Central and Copperbelt provinces were also less likely to have heard or seen any BCC messages about malaria, while urban residence was not associated with BCC exposure to malaria messages. Overall, these variables explained approximately 36% of the variance in the variable measuring exposure to BCC messages about malaria. Since the same regression model was used for both the propensity score matching and the treatment effect model, the coefficients from the model used to calculate the propensity score are described in lower panel of Table 2.

**Table 2:** **Biprobit regression model predicting whether respondent slept under a net the previous night (n = 3,263)**

	Coefficient	SE	p-value
**EQUATION B: SLEPT UNDER AN ITN LAST NIGHT**			
Number of nets in household (Ref = 1)			
2 nets	0.02	0.08	0.765
3 or more nets	1.16	0.08	0.001
Received malaria information from a health worker	1.18	0.06	0.001
Exposed to malaria information from BCC	0.48	0.12	0.001
Constant	-0.48	0.08	0.001
**EQUATION A: EXPOSED TO MALARIA INFORMATION FROM BCC**			
Age (Ref = 15–24)			
25-34 years old	-0.05	0.10	0.642
35-49 years old	1.34	0.11	0.001
Education (Ref = None)			
Primary	2.19	0.17	0.001
Secondary or Higher	3.94	0.19	0.001
Upper 2 wealth quintiles	-0.66	0.09	0.001
Province (Ref: Central, Copperbelt)			
Eastern, Northern, Luapula	-0.77	0.10	0.001
Lusaka	-0.28	0.16	0.089
Western, Southern, NorthWestern	-0.61	0.10	0.001
Has a child under the age of 6	-0.55	0.09	0.001
Lives in a district that received IRS	-0.39	0.10	0.001
Lives in an urban area	0.04	0.12	0.736
Constant	-2.19	0.21	0.001
**Correlation between residuals**	0.021	0.08	0.7791

Of the 3,380 women owning at least one ITN, 3,263 had complete information and were included in the analysis. Of these, 729 exposed respondents were matched with replacement to 2,498 unexposed respondents. For the 36 exposed respondents with the highest propensity scores, there were no unexposed respondents with propensity scores within the pre-determined proximity. These 36 respondents were dropped from the remaining analyses.

Table 3 compares the background variables between the exposed and unexposed respondents, both prior to and following the propensity scores matching. Prior to matching, all seven background variables differed significantly between these two groups. Once exposed respondents were matched to unexposed respondents with similar propensity scores, the two groups were statistically equivalent for all seven of these background variables.

**Table 3:** **Comparison of background characteristics between exposed and unexposed groups, prior to and following propensity score matching**

	% prior to matching (n = 3,263)			% following matching (n = 3,227)		
	Exposed	Unexposed	p-value	Exposed	Unexposed	p-value
Age						
15-24 years (ref)						
25-34 years	22	42	0.001	20	20	1.000
35-49 years	32	34	0.399	33	33	1.000
Education						
None (ref)						
Primary	36	52	0.001	36	36	1.000
Secondary or more	64	15	0.001	63	63	1.000
Province						
Central, Copperbelt (ref)						
Eastern, Northern, Luapula	30	45	0.001	30	30	0.955
Lusaka	4	13	0.001	3	4	1.000
Western, Southern, North-Western	35	35	0.853	36	36	1.000
Has a child under the age of 6 years	60	66	0.005	60	60	1.000
Upper two wealth quintiles	65	47	0.001	66	66	0.956
Lives in an IRS district	84	90	0.001	86	86	1.000
Lives in an urban area	28	17	0.001	28	28	0.953

### Treatment effect model

Table 2 presents the results of a two-equation treatment effect model. Equation A, which corresponds to the probit regression model used to calculate the propensity scores, regressed background variables on a variable measuring exposure to BCC messages about malaria. In Equation B, exposure to the BCC mass media messages, talked to a health worker about malaria, and the number of nets in the household were regressed on a variable measuring whether the respondent had slept under an ITN the previous night.

Use of an ITN the previous night was positively and independently associated with all three of the variables included in the model. Individuals exposed to BCC messages about malaria were more likely than those unexposed to BCC messages to have slept under an ITN the previous night. Similarly, individuals who had reported speaking to a health worker about malaria in the previous six months were also more likely to have slept under an ITN the previous night. Finally, respondents in households that owned three or more bed nets were more likely to have slept under an ITN than respondents in a household with only one bed net.

It is important to note that the residuals from Equation A and Equation B were uncorrelated. The correlation coefficient was equal to 0.022. The likelihood ratio test to assess whether this correlation was equal to zero had a Chi-square statistic with one degree of freedom equal to 0.079 and a p-value = 0.7791.

### Comparing exposed and unexposed groups in the two approaches

**Figure 1:**
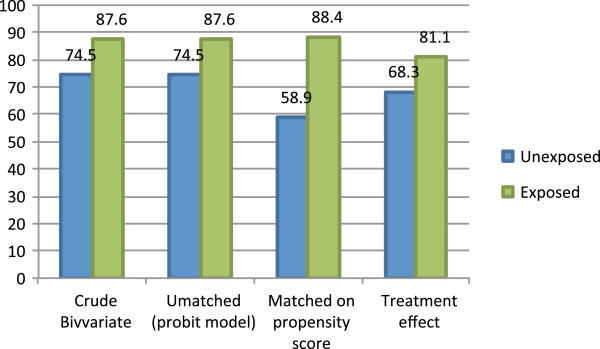
**Percent of respondents who slept under an insecticide-treated bed net the previous night, by exposure to the behaviour-change communication messages.**

Figure 1 compares the percent of exposed and unexposed respondents using an ITN the previous night under four conditions. First, the unadjusted condition presents the crude bivariate comparison of ITN use between the two groups. Second, the unmatched condition compares ITN use for the unmatched groups, controlling for the covariates in the probit regression model. Third, the matched condition compares ITN use for the matched exposed-unexposed groups formed using the propensity scores. Finally, the biprobit condition accounts for both the background variables used to form the propensity score while also controlling for the number of bed nets owned by the household and exposure to malaria information from the health worker.

In the unadjusted condition, exposure to the BCC messages was associated with a 16.2-percentage point difference in ITN use. Without controlling for any other variables, 87.6% of respondents exposed to BCC messages about malaria and 71.3% of respondents unexposed to messages reported sleeping under an ITN the previous night.

Prior to matching, comparing the exposed and unexposed groups while controlling for the covariates in the regression model results in a 13-percentage point different in ITN use, with 87.6% of those exposed using an ITN the previous, against 74.5% of those not exposed. After creating the matched groups on similar propensity scores, a 29.4-percentage point difference in ITN use is observed between exposed and unexposed respondents. Fifty-nine per cent of unexposed respondents reported sleeping under an ITN the previous night, compared to 88% of their exposed statistically equivalent peers. Finally, in the biprobit model, a difference of 12.7 percentage points is observed between exposed and unexposed groups, which also controls for the number of bed nets owned by the household and exposure to malaria information from health workers.

## Discussion

This analysis obtained different estimates confirming the positive effect of BCC message exposure on a woman’s use of a bed net the night prior to the survey from both the propensity score matching approach and the treatment effect model. Using either the unmatched regression model or the treatment effect model, a statistically significant effect of exposure to BCC messages on a woman’s use of a bed net to prevent the transmission of malaria was found, with a consistent effect size of around 12 percentage points. By contrast, the propensity score matching approach revealed a larger effect size of 29 percentage points, by creating statistically equivalent groups matched on their probability of being exposed to the BCC messages.

Although these findings were derived from an observational study rather than randomized clinical trial, confidence in attributing this effect to the BCC messages is warranted. The propensity score and the biprobit approaches provide unbiased estimates of the average treatment effects attributable to an intervention if the assumptions underlying the approach are valid. Since the two approaches used here rely on differing assumptions, the consistently significantly positive findings across both approaches provide convincing support for attributing a causal effect of BCC message exposure on bed net use.

The larger effect size of the propensity score matching approach may be due to the fact that it is based on the strong “ignorability assumption” (also known as conditional independence assumption), where assignment to treatment is assumed to be random. The inclusion of a variable measuring the number of bed nets owned by the household in the biprobit model, and its significant association with the outcome of interest, may have lessened the observed effect of exposure in that model. Similarly, its exclusion from the propensity score model may have resulted in an overly large estimate of effect of exposure in that model.

Differences in the observed effect size may also be due to the effects estimated from each model. The propensity score model estimates the average treatment effect on the treated, or ATT. The biprobit model provides an estimate of the overall average treatment effect (ATE), which combines the treatment effect on the treated and the treatment effect on the untreated. The ATT can be interpreted as follows: if the exposed respondents had not been exposed, the group’s mean ITN use rate would have decreased by 29.5 percentage points. Since the matched groups are statistically the same after matching, the matched result may be a better picture of the impact of BCC exposure. However, any unobserved differences between the exposed and unexposed groups may lead to a larger estimated ATT compared to the ATE. As Austin points out, the ATT is often of greater interest than the ATE for applied researchers [24], as it may be unrealistic to estimate effects on ITN use for an entire population.

This highlights the importance for understanding in advance the background factors that may influence exposure to a programme’s messages and including measures of these factors in the survey instrument. Both propensity score matching and treatment effect models are based on a regression model that predicts an individual’s exposure to the BCC messages. When this regression model is able to explain a substantial portion of the variance associated with message exposure, it decreases the likelihood that unmeasured factors associated with both message exposure and the outcome of interest will remain. Since it is these unmeasured factors that drive the correlation between the two residuals in the treatment effect model, the ability to condition exposure on all the important predictors of exposure should minimize the correlation between the residuals.

This study has some limitations. This type of analysis does not provide detailed information on what specific messages or channels may be more effective than others at achieving behaviour change. A second limitation is that the placement of the exposure questions in the women’s questionnaire prevents analysis by gender of the respondent. It is possible that message recall is different for men and women, and that effects on behaviour change could also be different. Further research is needed to explore the specific programmatic implications for behaviour change interventions.

## Conclusion

Despite these concerns, these analyses serve two purposes. First, they illustrate that BCC programmes can contribute to national programmes seeking to increase the use of ITNs inside the home. Second, these analyses offer a viable approach for evaluating the effectiveness of other BCC programmes promoting behaviours that will reduce malaria transmission or mitigate the consequences of infection.
